# It takes two to cleave: the logic of cluster assistance

**DOI:** 10.1101/gad.353586.125

**Published:** 2026-04-01

**Authors:** Owen Sanborn, Daniel Cifuentes

**Affiliations:** 1 Department of Biochemistry and Cell Biology, Boston University Chobanian and Avedisian School of Medicine, Boston, Massachusetts 02118, USA;; 2 Department of Virology, Immunology and Microbiology, Boston University Chobanian and Avedisian School of Medicine, Boston, Massachusetts 02118, USA

**Keywords:** DGCR8, Drosha, ERH, Microprocessor, SAFB, cluster assistance, microRNA

## Abstract

Mature microRNAs are generated in a series of sequential processing steps, creating multiple opportunities for regulatory bottlenecks. In this issue of *Genes & Development*, Shang and colleagues (doi:10.1101/gad.353316.125) dissect microRNA biogenesis by cluster assistance in human cells, demonstrating that ERH and SAFB2 have distinct functions in the processing of suboptimal hairpins. Beyond resolving the mechanistic dependencies on ERH and SAFB2, the study demonstrates that cluster assistance has been co-opted into a feedback mechanism to regulate DGCR8 levels and Microprocessor stability, elevating cluster assistance from a descriptive phenomenon to a physiologically integrated miRNA regulatory pathway.

## Abstract (precis)

In this Outlook, Sanborn and Cifuentes discuss a study in this issue of *Genes & Development* by Shang et al. that describes how Microprocessor cofactors ERH and SAFB2 play essential roles in the biogenesis of clustered suboptimal miRNA transcripts. Sanborn and Cifuentes reflect that cluster assistance not only serves as a defined pathway for miRNA production but may also serve as a regulatory feedback mechanism.

MicroRNAs (miRNAs) are small RNAs that regulate gene expression at the posttranscriptional level. The first committed step of miRNA biogenesis involves the excision of the stem–loop hairpin from the primary transcript and is catalyzed by the Microprocessor complex, minimally composed of DROSHA and DGCR8. The sequence and structural features of the primary hairpin are the key determinants of this first cleavage (Fang and Bartel 2015). In recent years, it has come to light that a cohort of accessory factors helps the Microprocessor complex when the primary miRNA transcripts do not fulfill these criteria. This is the case of cluster assistance, in which processing of a suboptimal hairpin is enhanced if it neighbors an optimal hairpin on the same transcript (Truscott et al. 2016; Fang and Bartel 2020; Shang et al. 2020; Shang and Lai 2023). Previous works identified ERH and SAFB2 as auxiliary proteins enabling cleavage of suboptimal primary miRNA hairpins via cluster assistance (Fang and Bartel 2020; Hutter et al. 2020; Kwon et al. 2020; Jang et al. 2025). Genetic ablation of either factor specifically depletes clustered suboptimal miRNA abundance, suggesting that they work in tandem, yet the hierarchy of molecular events leading to the processing of suboptimal Microprocessor substrates remains unexplored. Additionally, it is not clear whether cluster assistance is an additional nuance of miRNA processing or an active regulatory checkpoint controlling miRNA biogenesis. In the present study, Shang et al. (2026) set out to identify the distinct roles of ERH and SAFB2 and attempt to place them within a sequence of events for cluster assistance with a functional outlook.

Through a tour de force of targeted knockout and knockdown of Microprocessor cofactors, Shang et al. (2026) reveal that these factors act hierarchically, as SAFB2 can partially rescue ERH deficiency, whereas ERH cannot compensate for the loss of SAFB2, showing their distinct roles as well as a stronger functional dependence on SAFB2. This greater reliance on SAFB2 is reinforced by tethering experiments in which λN-DGCR8 is directed to isolated suboptimal miRNAs via a BoxB motif. Under these conditions, ERH becomes dispensable, but processing still requires SAFB2. These results are in contrast to another study (Jang et al. 2025), where DGCR8 tethering led to an ERH—but not SAFB2—dependency, albeit differences in the BoxB configuration and loss-of-function approaches may explain the mechanistic differences.

Another point of contrast between the studies by Shang et al. (2026) and Jang et al. (2025) is whether Microprocessor must physically interact with ERH or SAFB2. However, this is challenging to resolve experimentally, because DGCR8 overexpression can mask ERH dependency and obscure the contribution of specific interaction surfaces. Altogether, data from both works support a hierarchical model in which Microprocessor is first recruited by an optimal hairpin and then transferred to cleave a structurally weak substrate by the combined yet distinct roles of ERH and SAFB2. However, Shang et al. (2026) refine this framework further, proposing that ERH functions specifically in the transfer of Microprocessor between adjacent hairpins, while SAFB2 acts directly at the suboptimal hairpin to promote stable recognition and cleavage. Future structural work will complement the models that emerge from these genetic and biochemical insights.

A genome-wide profiling of miRNA processing in ERH and SAFB1/2 mutants after metabolic labeling conducted by Shang et al. (2026) further clarifies the relevance of each cofactor in cluster-assisted miRNA processing. SAFB1/2 depletion causes far broader and stronger defects in miRNA accumulation than ERH loss, again identifying SAFB2 as the dominant effector of cluster assistance. As anticipated, these defects selectively affect clustered and structurally weak miRNAs. Importantly, combined depletion of ERH and SAFB1/2 does not exceed the effect of SAFB loss alone, consistent with the rescue experiments’ results. Moreover, when Microprocessor is ectopically overexpressed, the requirement for ERH diminishes, while SAFB2 remains essential. Collectively, these results position SAFB2 as the principal driver of suboptimal miRNA cleavage.

Any additional cofactor or processing step creates an opportunity for regulation. In the case of cluster assistance, this is illustrated by the autoregulation of Microprocessor via a conserved suboptimal hairpin, miR-1306, embedded within the coding sequence of the DGCR8 transcript (Han et al. 2009). When DGCR8 levels are high, Microprocessor cleaves both optimal miR-3618 and the adjacent suboptimal miR-1306 in a SAFB2-dependent (Shang et al. 2026) or ERH-dependent (Jang et al. 2025) manner, resulting in reduced DGCR8 expression and reduced Microprocessor abundance. As Microprocessor activity declines, cleavage of miR-1306 becomes increasingly inefficient, permitting DGCR8 levels to recover and restore Microprocessor capacity. Importantly, the DGCR8/miR-1306 autoregulatory circuit illustrates that Microprocessor abundance is limiting and tightly controlled. This constraint favors local processing strategies that route scarce processing capacity toward selected substrates. Overall, these results from Shang et al. (2026) and Jang et al. (2025) elevate cluster assistance from a descriptive phenomenon to a mechanistically defined and physiologically integrated pathway, with broad implications for miRNA biogenesis and its regulation.

While the DGCR8/miR-1306 feedback circuit illustrates how structural suboptimality can serve as a homeostatic regulatory feature, for other miRNAs, cluster assistance is a mandatory response to different evolutionary pressures. miR-451, the archetypal substrate of cluster assistance (Fang and Bartel 2020; Shang et al. 2020), underscores this point. Its unusually short hairpin has evolved for Ago2-dependent maturation, and cluster assistance functions not as a regulatory checkpoint but as a necessary accommodation that allows processing of miR-451 under conditions where Dicer-dependent miRNAs are suppressed (Kretov et al. 2020). In this context, ERH and SAFB2 broaden Microprocessor's capability to process substrates that feed into distinct downstream pathways. A key challenge for future work will be to distinguish cases in which cluster assistance is imposed by downstream noncanonical processing requirements from those in which suboptimality has been repurposed as an opportunity for additional regulatory control.

In summary, Shang et al. (2026) establish cluster assistance as a defined pathway with different dependencies for ERH and SAFB2. Beyond enabling steady-state production of suboptimal miRNAs, this study, together with the study by Jang et al. (2025), shows that cluster assistance is integrated into a feedback mechanism that regulates DGCR8 levels, elevating it from a descriptive phenomenon to a physiologically embedded regulatory pathway.
